# Positioning Phytosanitary Food Treatments: Exploring the Role of Business-to-Consumer Stakeholder Literacy as an Information Gatekeeper in New Zealand

**DOI:** 10.3390/foods11142108

**Published:** 2022-07-15

**Authors:** Denise M. Conroy, Jennifer Young, Amy Errmann, Tracey Phelps

**Affiliations:** Department of Stakeholder and Consumer Intelligence, The New Zealand Institute for Plant and Food Research Limited, 120 Mount Albert Road, Mount Albert 1025, New Zealand; denise.conroy@plantandfood.co.nz (D.M.C.); jenny.young@plantandfood.co.nz (J.Y.); tracey.phelps@plantandfood.co.nz (T.P.)

**Keywords:** public health, food marketing, health literacy, business-to-consumer, phytosanitary treatments, global food systems

## Abstract

Various phytosanitary treatments are used globally to ensure biosecurity for borders, whilst maintaining public health and safety in the consumption of fruits and vegetables. However, public health literacy of phytosanitary treatments is still low. Furthermore, little is known of the literacy on important information gatekeepers, such as business-to-consumer (B2C) stakeholders. This study investigates the health literacy of phytosanitary treatments by B2C stakeholders, and the subsequent positioning marketing narratives as an outcome of such literacy. We use health literacy as a theoretical lens for classifying different strategies that B2C stakeholders may use when positioning phytosanitary food treatments. Data were collected using in-depth interviews with 12 purposefully recruited New Zealand B2C retailers, based on the criteria of making and/or influencing decisions about the supply of fresh fruits and vegetables to consumers. Thematic analysis was used to analyze the qualitative data. The study advances research in food marketing by showing how different literacy levels may influence marketing narratives in the global food system. It makes a valuable contribution to literature by unveiling how appraisals of invasiveness, familiarity, naturalness, and sustainability lead to different applications of positioning narratives: the purist approach, maintaining the romance, and full transparency.

## 1. Introduction

An increasingly important issue in global public health is health literacy. Health literacy refers to the interactions between stakeholders (e.g., consumers, retailers, public health officials) and health information (e.g., new food technologies, nutrition information, food safety) to subsequently support informed decisions concerning health [1]. Literacy is important, as research shows that health information is becoming increasingly complex, and thus stakeholders must have fluent knowledge to make informed decisions, in order for them to make better judgements [2]. Literacy often influences a stakeholder’s knowledge of health information [3], can lower fear-based judgements [4], and can increase trust towards health information [5]. One important factor facing health literacy is in understanding and evaluating health information about novel food technologies when making food choices [6].

An important novel food technology that represents a global solution to health and safety needs is X-ray irradiation. X-ray irradiation, which uses ionizing radiation from X-rays, represents one of many phytosanitary treatments that protect consumers from foodborne illnesses [7], as well as reducing biosecurity threats [8]. Other phytosanitary treatments include chemical treatments (e.g., methyl bromide fumigation) or high pressure washing (e.g., rinsing fruits and vegetables at high temperatures/high water pressure) [9]. In New Zealand, produce (fruits and vegetables) is the category treated with irradiation, making up approximately 8% of this category [10]. Irradiated food must be labelled to state if the food or any of its ingredients has been treated with ionising radiation [11]; however, there is no mandate to include a Radura logo on the package [12]. As the total amount of irradiated food in New Zealand is low, and there is no current legislation mandating international symbols such as Radura, consumers’ familiarity with irradiation may be minimal [13]. Against the backdrop of low familiarity, one major point of contention is the possible low health literacy rates of stakeholders and the public towards technologies such as irradiation. Further, stakeholders with perceived low literacy may not be aware that methyl bromide contributes to ozone depletion [14], while in contrast, those with perceived high literacy may have concern over methyl bromide as it contains chemicals which remain as residues on treated food [15]. Phytosanitary treatments represent a global challenge to health literacy: phytosanitary treatments are important for indicating regulatory protective treatments, processes, or ingredients to stakeholders [16]; however, low literacy of such treatments may lead to unclear perceived increased health risks in the public [13].

When it comes to health literacy issues within the food system, business-to-consumer (B2C) organizations are often the gatekeepers of health information before it makes its way to consumers [17]. The confluence of B2C stakeholders’ own health literacy in conjunction with phytosanitary technology represents a unique discourse. Food systems globally need phytosanitary measures to protect biosecurity at borders and consumers from foodborne illnesses [18,19]; however, not all B2C stakeholders are equally literate in the differences of such treatments [10,20]. Thus, we introduce the integration of health literacy as a lens through which B2C stakeholders’ literacy may influence the information (e.g., positioning strategies) of phytosanitary treatments to consumers. In doing so, we build on research which explores the integration of health literacy into food systems [1], discussing how B2C literacy results in different judgements on phytosanitary treatments, and ultimately, how such treatments aim to be marketed to consumers.

The current research makes three distinct contributions. First, we help clarify how different levels of health literacy of B2C stakeholders inform strategies, impacting how health information is disseminated, marketed, or accessed [1]. Second, we highlight that literacy is broadly important to food marketing, and more specifically to the understanding of phytosanitary treatments, as health information is becoming increasingly complex and consumers need clarity to make better judgements [2]. Finally, we build on research which explores the integration of health literacy into food systems. Such integration is important as various literacy levels result in different judgements. The overall goal is to contribute knowledge to global public health research regarding the way health literacy impacts on the understanding of phytosanitary health information. The paper begins with an overview of literature in the domains of health literacy, phytosanitary awareness, and literacy gatekeepers. Subsequently, the materials and methods section covers data collection and the thematic analysis. Next, our findings from the interviews are discussed in three distinct themes that correspond to the [1] health literacy model. Finally, we conclude with a discussion of implications, limitations, and future research.

### 1.1. Health Literacy

At a functional level, health literacy involves access, comprehension, and judgement of information to enable making informed decisions about health [21]. For instance, accessing nutrition labelling can help consumers identify foods high in saturated fats, sodium, or sugars [1]. On an interactive level, health literacy requires cognitive skills to comprehend, and thus evaluate, health information [22], while on a critical level it encompasses judgement skills to address barriers to healthy decision-making [23]. Thus, health literacy ultimately rests on regulators of food systems to provide good-faith health information for stakeholders to access and evaluate, which also places a responsibility on stakeholders to correctly appraise and judge information.

However, health information is consistently reported as difficult to understand [24], making food or health decisions is therefore an increasingly complex task [1]. Specific to this research, literacy of phytosanitary procedures remains an area unknown to B2C stakeholders who are exposed to differing amounts of phytosanitary information, and furthermore, responsible for the dissemination of its related information to consumers. Specifically, we adopt the notion that health literacy relies on “knowledge, motivation and competencies to access, understand, appraise, and apply health information in order to make decisions concerning… health promotion” [25]. By understanding how stakeholders *access*, *understand*, *appraise*, and *apply* phytosanitary information, we may more effectively enable B2C enterprises to become more health literate at functional, interactive and critical levels [4].

### 1.2. Phytosanitary Awareness and Literacy Gatekeepers

The need for efficient and safe biosecurity processes to control invasive pests arising from the international trade of fresh fruits and vegetables remains a high global health priority [19]. However, one major point of contention is that stakeholders may have low awareness of phytosanitary technologies and may be fearful of these technologies [17]. For instance, consumers associate X-ray irradiation with harmful radiation, while in contrast, X-ray may be a solution that is most compatible with solving sustainability and public health challenges [14]. Such low awareness is indicative of low literacy levels, and subsequently causes stakeholders to be less trusting and have lower acceptance in emerging food technologies [18], such as phytosanitary treatments. Traction is needed to raise literacy levels in key players within the public.

Improving literacy regarding phytosanitary treatments begins with access to information. Whether New Zealand stakeholders, including B2C enterprises, will accept X-ray as a safe and appropriate replacement phytosanitary treatment remains an important consideration for the New Zealand Government, and for consumers reliant on the safety of fresh produce. A key consideration in the adoption of X-ray irradiation, amongst other phytosanitary treatments, is the gatekeeping of health information which can be made available. Whilst consumer studies across multiple countries indicate continued negativity towards food irradiation [2,17,26], little research has considered the perspectives and role of B2C retailers in this overall literacy process. An understanding of B2C stakeholders would help determine the judgements, appraisals, and strategies of different literacy levels needed to communicate adoption of phytosanitary technologies.

## 2. Materials and Methods

### 2.1. Recruitment and Data Collection

Twelve interviews were conducted in April 2021, using Zoom, the cloud-based virtual meeting platform. Participants were purposely recruited [23] by a specialized recruitment provider who were briefed to recruit from a list of New Zealand B2C retailers. We structured the purposeful recruitment of participants on the criteria of making and/or influencing decisions about the supplying of fresh fruits and vegetables to consumers [27]. These included senior personnel from supermarket chains, procurement managers from leading meal-kit delivery companies, and business owners. All participants were based in Auckland, although in several cases their businesses operated nationally. Participants received a ‘Participant Information Sheet’ and signed a ‘Consent Form’ prior to their interview. Each participant received an NZD 100 cash payment as a token thank you gift for their participation. The study was developed in accordance with approved ethics protocols for participants at the New Zealand Institute for Plant and Food Research Limited and ethical approval (ref: P/321079/05) was obtained before the commencement of the data collection (15 April 2021).

**Figure 1:**
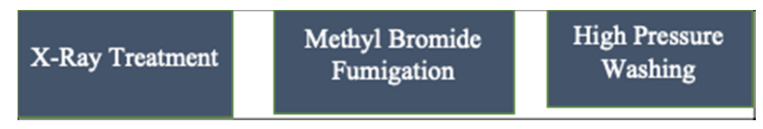
Methods for Phytosanitary Treatments.

The interviews, which were audio-recorded, were moderated by one researcher according to a semi-structured interview guide that was created using current literature [26] on phytosanitary treatments [17]. Participants were firstly asked about their awareness of different methods of treatments and then introduced to X-ray treatment as a replacement option for methyl bromide fumigation. Participants were also asked to consider and compare three treatment methods (see Figure 1) and comment on how they accessed information, understood, appraised, and applied knowledge in relation to each technique [28]. This enabled the researcher to assess the stakeholders’ literacy of each treatment [1].

Another researcher attended the interview, made notes, and asked supplementary questions where needed. The interviews lasted approximately 45 min. The aim was to explore each stakeholder’s literacy of phytosanitary treatments, with specific attention paid to X-ray irradiation, as it is one of the most important future phytosanitary treatments to be used globally [16].

Participant profiles are presented in Table 1. Where possible, the recruitment company attempted to provide participants with a variety of levels of seniority, type, and size of companies. Age, gender, and cultural backgrounds were not specified as part of the purposive recruitment, although data were obtained in regard to these characteristics.

### 2.2. Data Analysis and Interpretation

All interviews were transcribed using the Otter Ai software. Otter is a voice recorder that offers automatic transcription, which allows for the capture of long-form conversations among multiple people. After cleaning and ensuring accuracy of the transcripts, the analysis process commenced. NVivo was used to store the transcripts and facilitate the coding process. Three researchers used an iterative, data-driven qualitative approach to identify and analyze major themes emerging within the data [29]. The analysis began with a reflection and discussion after the interviewing phase [6,24]. The researchers then worked closely together identifying and contesting patterns and collaborating on definitions of codes, using open coding to identify the general patterns derived from the texts of collected transcripts [6,29].

Following the back-and-forth process of open coding, recurring patterns and more definitive themes were then axial coded to identify the meaningful relationships among the open codes across the data [5,28]. Axial coding is the technique to make comparisons and identify meaningful relationships among the open codes, across different questions within the entire dataset. This enabled us to identify overarching themes based on primary patterns from the open coding process, giving a more holistic understanding of the wider topic related to the research questions. Coding was consistently reviewed and discussed among the wider research team to gain triangulation [3]. Throughout the analysis process, verbatim illustrative quotations were noted. Where participants’ comments are quoted in this report, participant numbers are used to ensure anonymity [30].

## 3. Results

The findings from the interviews are discussed below in three distinct themes that correspond to the [1] health literacy model. Each theme highlights rich descriptions of how participants accessed/comprehended, appraised, and applied knowledge [31] in relation to phytosanitary treatments. Specifically, the themes include: (1) Access and Comprehension, (2) Phytosanitary Appraisals, and (3) Application Narratives for Phytosanitary Positioning. See Table 2 for a conceptual map.

### 3.1. Access and Comprehension of Phytosanitary Methods

Participants were asked to consider fresh fruit and vegetable produce and their access to information and knowledge of possible phytosanitary treatments. Views ranged from those with only superficial associations about the physical processes to those with very high levels of knowledge.

#### 3.1.1. Superficial Associations

X-ray irradiation treatment was introduced to participants as a method of low-beam food irradiation that was already available as a treatment in some countries and was now being considered for New Zealand as a phytosanitary treatment. For participants with only a superficial awareness of what X-ray phytosanitary irradiation was, there was a tendency to link ‘X-ray’ with radiation.

*“A lot of people believe… if they don’t fully understand what that treatment might be doing to their food in the long-term, they would probably rather not have it, and you know, especially some of our customers are maybe recovering from a cancer or cancer treatment. And they want to just eat as clean and as healthy as possible. I’m sure if they heard it was radiation or something, they probably wouldn’t want to have much to do with it”*.

[P7, General Manager, Premium Organic Supplier]

The words were discussed as having negative and possibly frightening connotations for consumers because of their link to radiation. Words such as ‘poison’, ‘toxic’, ‘nuked’, and ‘zapped’ were used.

*“Well, I mean everyone I guess has been for an X-ray. You know, they put on your protective gear and then they say, look out, and they all rush out of the room and leave you siting there. So, in terms of safety and food, you’re probably not that keen on it. It does have connotations. I guess from an academic perspective, but if that’s what someone has on their mind then they will be concerned”*.

[P6, Business Owner, Organic Supplier]

Some participants stressed that associating X-ray with radiation could misfire badly and cause consumer backlash/unintended consequences that could be magnified by access to media.

*“If it comes solely from a single source, you’re going to have people that will argue it’s coming in mainstream media… If it’s pushed by the alternatives, you’ll get the mainstream saying it’s a conspiracy”*

[P7, General Manager, Premium Organic Supplier].

#### 3.1.2. Trust in Regulations

For other participants, there was a tendency to accept that phytosanitation was a necessary process that happened, and that regulatory bodies would ensure controls in a safe way. In this way, there was belief that the responsibility to understand information would be put on the regulatory bodies, rather than on the retailors themselves to comprehend.

*“Not specifically aware. I know that it would be treated somehow. The how and the details, I’m not sure… That sort of thing is way above my paygrade. So, I tend to think if it’s been done by the New Zealand authorities… If it’s something that’s approved by New Zealand… I’d be happier with that”*

[P10, Small Business Owner, Catering].

Further, some participants voiced that it may be easier to rely on regulating authorities to have knowledge about phytosanitary, rather than ask questions or critique themselves.

*“I guess, the people that bring it in, don’t want to tell people or either… some people just assume it and don’t really want to know much. Because I don’t know, I guess it’s one of those trade secrets, right? It gets in here. People don’t really ask too many questions”*

[P8, Retail Manager, Food Service].

*“So here nobody asks for that information. And they didn’t provide that information. So, there’s nothing there. I think there is nobody putting out that information. Maybe because they’re scared of, okay, if we put everything out there for our customers to see, and then the customer is less likely to buy that product. So, there’s nobody doing any efforts on that kind of thing to tell the customer”*

[P2, Produce Category Manager, Premium Supermarket Chain].

#### 3.1.3. Justified Safety

Some participants with a higher level of knowledge about phytosanitary treatments justified that methyl bromide-treated produce was safe for customers, because there was no alternative.

*“I’m aware of the treatments, so I’m not going to say them out loud… Well I mean, they are not very nice to be fair… well I mean the irradiation treatment, probably as much as people hate to hear that word, it’s probably the safest treatment that we’ve got”*

[P11, Business Manager, Supermarket Chain].

Confidence in safety across the New Zealand fruit and vegetable food system stood out clearly. A strong preference for New Zealand produce was driven by participants’ awareness of the country of origin. Further, it seemed there was an acceptance that any chemical/pesticide treatment by growers would be safe and relatively benign.

*“When you think of New Zealand, you think of the wholesomeness. You think of… you tend to think of trust. You think, oh, my grower is going make sure that the pesticide he is using on this product, it’s good because we have those laws in place, and we have those regulations. Yeah, I think consumers trust”*

[P3, Procurement Manager, Meal Kit Delivery].

*“That’s the number one thing for us, for this country, you know, we want to be more environment friendly. So, this is a great way to do it. I think the product of will be perceived better because it’s just X-ray treated”*.

[P2, Produce Category Manager, Premium Supermarket Chain]

When it came to accessing information, those with higher literacy (Table 2) recommended government handling of information, owing to the fact that the public would rely on health information from the government, because of increased trust from the global pandemic.

*“I think now’s probably the perfect time to have a government message that people trust. Because a lot of people are trusting of the government after our handling of COVID… the government has said this is safe and we back them up and we are supporting of that”*

[P4, Ingredient Coordinator, Meal Kit Delivery].

### 3.2. Phytosanitary Appraisals

When asked about how participants appraised X-ray, methyl bromide, and high pressure washing, participants voiced distinct judgements in relation to their literacy levels. Participants who seemed to have lower levels of literacy voiced that phytosanitary treatments, in particular X-ray, sounded invasive. Participants with some level of literacy discussed worries about the familiarity or risk to perceived naturalness of food, and those with a high level of literacy discussed scientific advantages of sustainability.

#### 3.2.1. Invasiveness

Some participants selected X-ray treatment and methyl bromide fumigation as being perceived as similar by consumers because of their more invasive nature, in contrast to the more natural connotations of using water.

*“I think just instantly the water one is probably the most appealing. Just for the sake of less chemical involvement. I guess, or less of a treatment… water is probably something they would be comfortable with”*

[P7, General Manager, Premium Organic Supplier].

Further, appraisals of X-ray ‘sounding’ invasive were used to justify a lack of interest in the method.

*“I think the word irradiated because it’s got “radiated” straightaway, it resonates badly”*

[P11, Business Manager, Supermarket Chain].

Finally, some regarded X-ray or methyl bromide fumigation to be an intrusive process, questioning any residual effects that may linger in the food.

*“Yeah, everything to do with X-rays that you know, and what is residual in a product. If you look at, you know, phyto-plastics and other, which just comes in from sitting in a packet. If you’ve gone through an intrusive process like the X-ray or irradiation, then what is the residual? And if we’re working hard to get good clean product, what’s the, you know the outcome of going through that process? It’s the same with fumigation. With X-ray, what’s happening to that product?”*

[P6, Business Owner, Organic Supplier].

#### 3.2.2. Familiarity

Some participants mentioned methyl bromide might stand out because it has been around for many years, and some consumers would have heard of it being used previously. In this way, it was more of a familiar treatment. Other concerns arose from the lack of familiarity of X-ray.

*“I don’t know… It’s hard because the methyl bromide sounds like… it’s tangible, it exists, and you can see it, you know, it’s there and you can... I don’t know? I feel like consumers can maybe understand that better. Whereas X-ray treatment: what does that mean? You know? But it’s got no bones, how did you take a picture of that? Like what is that? But then you think, oh pesticide, or like, you know, fumigating nasty bad things. X-ray, oh, medical, safe enough for people to get their bones X-rayed. Maybe it’s better… and it obviously sounds like it’s going to be better for the environment, but is it going to be as safe or safer for the consumer? I don’t know”*

[P4, Ingredient Coordinator, Meal Kit Delivery].

*“If you’re choosing between X-ray and fumigation, there would need to be an education process to explain what it is. Just off the top of my head right now. People know it’s fumigated, they might have seen it before, I can live with that, but if it’s X-ray, I have no idea what it means. I’m going to have to do some research and ask the question, and probably be a bit more hesitant about that product would be the view right now”*

[P6, Business Owner, Organic Supplier].

Some participants voiced that their customers were more familiar with organic products, as being organic was perceived to be a treatment in itself. In this way, if a fruit or vegetable was treated by a phytosanitary method, it would not be considered as familiar as organic.

*“There are a portion of our customers who believe strongly in organic based as part of a treatment, you know, there is that saying that the food you eat can be medicine or poison over a period of time. A lot of people believe that strongly, and if they don’t fully understand what that treatment might be doing to their food in the long term, they would probably rather not have it, and you know, especially some of our customers are maybe recovering from a cancer or cancer treatment. And they want to just eat as clean and as healthy as possible. I’m sure if they heard it was radiation or something, they probably wouldn’t want to have much to do with it”*

[P7, General Manager, Premium Organic Supplier].

However, as participants became more familiar with phytosanitary treatments, they indicated that there may be more adoption of phytosanitary methods, as long as consumers became familiar with them. This suggests that with increased familiarity, there may be an increase in literacy levels.

*“I don’t really think that it resonates with them unless it’s highlighted. I mean, when we first bought the mangoes in. And that was the first irradiated fruit we bought into the country, there was a bit of a roar around them, but it soon dropped down when they had such beautiful, beautiful big mangoes. Mangoes better than they’ve ever had before because we can import those from other countries. It all vanished because they wanted some more of those mangoes, but they are irradiated, oh that doesn’t matter, they still wanted more of them. So, it does vanish. I think, it will be how it’s communicated, if and when we get there because no one says much about methyl bromide, do they? But it’s been going on for years”*

[P11, Business Manager, Supermarket Chain].

#### 3.2.3. Naturalness

Some participants were not aware of other potential benefits of the use of X-ray (such as shelf-life extension, sprout inhibition), but when these were discussed, retailers felt that these benefits could also be perceived as unnatural by consumers as they value ‘fresh food’, and these practices would seem contrary to this.

*“I would like to see the science and understand it before I can comment properly. A little part of me, you know, the non-natural process that’s having to be applied. I’d be… not wary of it, but I’d just want to understand it better before I could clearly comment. But if science does show it to be a safe alternative with lesser impact from an environmental and health perspective then I think… I’m all for technology as well. You know, find better ways of doing things that can improve circumstances with little negative downsides”*

[P7, General Manager, Premium Organic Supplier].

Other participants regarded any added phytosanitary processing as unnatural, commenting that the more processing is carried out on food, the more it strays from the original composition of the food. Therefore, they believed they were highly literate about what ‘natural’ was, and that phytosanitary treatments may stray from that.

*“Any systems or any processes that are required that aren’t natural… I would believe there’s likely to be some side effects, negative, somewhere. Whether that’s from a health perspective, an environmental perspective, a cost perspective, emissions perspective, you know, anything that isn’t just a natural process”*

[P7, General Manager, Premium Organic Supplier].

*“I’m not really 100% in having any treatment or anything. I would rather have it natural”* [P1, Fresh Produce Buyer, Large Supermarket Chain].

#### 3.2.4. Sustainability

Respondents who had higher levels of literacy, in that they had more knowledge about the intricacies of methyl bromide using chemical fumigation, were very open to accepting and adopting X-ray irradiation.

*“Anything to do with chemicals, I think people will object in the future too… If they know this is a safer procedure, and it’s also good for the environment. That, I think, will definitely be the people’s choice… And we use X-ray even personally and I confidently can say that the vast majority of the population would rather have it X-rayed than being treated by chemical… health wise… the number one thing is what you put in your body is what you become. So, if they know that any amount of this chemical might be going into their bodies. That is a red flag. I think for anybody”*

[P9, Procurement and Project Manager, Food/Juice Outlet Chain].

*“I think if we were totally honest and we said to people, this is what’s happening to the bananas that you’re getting in the box today, they’ve been flushed with a poisonous gas, or they’ve been scanned with an X-ray, I think most people would pick the X-ray”*

[P12, General Manager, Large Food Service].

Further, sustainability judgements were made based on what participants knew about the environmental impacts of phytosanitary methods. Participants needed some form of literacy to make these judgements.

*“Growing up as a kid in the 90s. I was very aware of CFCs, and their effect on the ozone layer. And you know, we were getting to a point where, we are seeing, oh look the hole in the ozone is slowly closing. We’re at risk of delaying that or reopening that because we’re using this thing to fumigate our food when there’s an alternative… Which is an X-ray, which we currently use to, you know, check our teeth and our bones and stuff, and if it’s controlled”*

[P5, Small Business Owner, Food Outlet].

*“I think it’s a great way to tell our customers a story, you know, this is something not fumigation and it’s definitely better for our environment, you know”*

[P2, Produce Category Manager, Premium Supermarket Chain].

*“I would say I would lean more towards the X-ray, just because I feel like it will probably have no impact on the flavor or quality of the produce or ingredient that’s coming in. And then if I think about the information that you said about the fumigant not being great for the environment. Well, I’d say that probably aligns better with what we’re trying to do”*

[P4, Ingredient Coordinator, Meal Kit Delivery].

### 3.3. Application Narratives for Phytosanitary Positioning

When it came to strategies about the narratives to employ, three contrasting perspectives were evident. The perspectives differed according to the perceived role of literacy, and whether products that were treated seemed more invasive—calling for a purist approach to keep information in the dark—to a naturalist or familiarity approach and the desire to maintain the romance of natural narratives—to the understanding that there were benefits to phytosanitary measures, in which participants maintained full transparency in their narratives.

#### 3.3.1. The Purist Approach: Keep in the Dark

Some participants indicated that they would prefer to limit their range to New Zealand produce to avoid the use of phytosanitary treatments over concerns of the potential negative health implications. This arose from a strong need to protect the health of their customers who have low literacy about what they are ingesting.

*“Are we better just to say, hey look, actually that’s not available. And a lot of people in organic spaces actually go, I’d rather not have it. I’ll stick to what I get locally and in season. And then I’d suggest that we would probably stick to local or source the product from countries where the irradiation and other, wouldn’t be required… I would change our sourcing methodology specifically”*

[P6, Business Owner, Organic Supplier].

*“There will be a certain amount of people that no matter how you dress it up, they won’t be happy with any treatment on fruit, whether it’s been fumigated with methyl bromide or irradiated, or heat treated. You know, we used to import stuff from Fiji that was heat treated. And people just don’t want to think that fruits have been handled in those ways. They just want it to be as it was grown”*

[P12, General Manager, Large Food Service].

Several participants suggested softening the language in narratives to reduce the negative and ‘scary’ connotations.

*“Most people are willing to embrace new technology, and you’re always going to have a few people that resist it. I just think we’re in a stage where due to social media and all that, we’ve got a few more kind of stragglers and I guess people who will look for alternative facts. And they will use the long words to scare themselves and other people. And so, it really needs to kind of be simplified I think, I guess, green wash… call it something else, you know, make it sound less scary and people might adopt it”*

[P5, Small Business Owner, Food Outlet].

#### 3.3.2. Maintaining the Romance

Informing consumers about phytosanitation processes was viewed as a complex narrative for consumers to understand. Those in the traditional supermarket model assumed consumers would be concerned and/or react negatively to both methyl bromide and X-ray treatment, particularly if they did not know these treatments were going on behind the scenes. Those in higher positions and with more knowledge of importation processes saw an advantage in maintaining the status quo to avoid entering a complex area that could potentially destroy the perceived ‘romantic ideal’ of fruit and vegetable growing. It was made clear that whilst they recognized that regulatory authorities carried out a good and necessary job, the process of phytosanitation was best kept to as low a profile as possible.

*“But you know, you wouldn’t certainly say that you’re using methyl bromide or something like that to the public because that would really scare them off… You’ve got to be careful what you highlight because you know, people will start to get unhappy about the whole thing. But it’s one of those things that’s going to be… I’m not sure how it would go”*

[P11, Business Manager, Supermarket Chain].

Several retailers alluded to the romantic ideal customers held for fresh produce, and wanted to maintain the positive ‘halo’ that surrounds the sector.

*“Bananas and things… it’s not a grubby business but it’s a complicated business. And lots of things happen to that fruit from harvest to arrival. And if the people knew everything that goes on, and the length of time it sits on a boat, or cool stores being gassed, being heated, being fumigated, and being ripened. People probably wouldn’t be so keen at all. People in their heads like to think that fruits picked off a tree by a happy smiling person, put in a basket and taken to the market type of thing. But that’s just not how it happens. We like to think that”*

[P12, General Manager, Large Food Service].

It was important that labelling practices were in place, so that the company would not be seen to be hiding the process, as it is not something they can influence.

*“What it has done to the fruit. What the effects are? Is the food safe to eat? That’d would be the obvious thing of course. But in theory it’s been done to make the food safe to eat. But I think it could bring up that question of, well, that’s fine, but methyl bromide doesn’t sound that great. And everyone knows that X-ray is radiation. So, I can see them certainly questioning it, as to whether it’s safe to eat. And I’m not going to be qualified enough to give them the answers to that, to be perfectly honest. All I can say is, well, it’s labelled here, and it’s says fit for human consumption”*

[P10, Small Business Owner, Catering].

The overall implication was that for the broader consumer market who buy on price, the most effective method was warranted.

*“I think for me as a retailer I think it won’t concern me... because I’m putting whatever is available, because there are no other mangoes there. If somebody wanted to buy or eat mangoes, that’s the only option”*

[P2, Produce Category Manager, Premium Supermarket Chain].

#### 3.3.3. Full Transparency

Participants with business models that more overtly incorporated sustainability, displayed a highly literate approach. They were more likely to express a felt level of responsibility and implication in the process, and wanted to be seen as trusted and in line with the values of their consumers. They indicated that their consumer base would seek out and evaluate information from many sources for themselves about the safety of various methods and then make an informed decision.

*“I feel like if we give our consumers enough information about that process, I think they would be fine. You know, especially with the sustainability initiatives that we’re going through at the moment. You know, to say, this is what’s happening to the environment if we’re using this. So, as a company we’re looking at moving into the X-ray, which is better for biosecurity and everything… I think they would be okay. It’s just about arming our customers with that information”*

[P3, Procurement Manager, Meal Kit Delivery].

Greater education for consumers was seen to be one way of dealing with the potential conflict over the need for biosecurity and the desire to consume products that are imported. Interestingly, one organic supplier saw a need for balance if we are to move organics more to the mainstream, thus X-ray could be seen as compatible with organic produce.

*“But if science does show it to be a safe alternative with lesser impact from an environmental and health perspective then I think… I’m all for technology as well. You know, find better ways of doing things that can improve circumstances with little negative downsides. I think our customers put a lot of trust and faith in us, to do that work for them. And so, if we said to them, we were confident, and comfortable. I think a lot of them would be okay with it”*

[P7, General Manager, Premium Organic Supplier].

For others, there was a realization that organics within New Zealand had not reached a tipping point of larger volumes owing to the New Zealand produce ‘halo’.

*“I think there’s also a perception that New Zealand is beautiful and green and lush. So, I think people just almost visually assume that everything is growing really well, and they probably don’t see the disparity between organic and conventional as clearly. Whereas if you go to some other places where the practices are… dire. Like, you can see something that has been farmed organically versus not, and the land is almost dead versus not”*

[P7, General Manager, Premium Organic Supplier].

However, how much a consumer may be motivated to access information that was available was based on differing demographics and consumer needs.

*“For cheaper brands, I would say they would be the least concerned with this. They’re not as far as we can tell, big label readers. They’re not looking at the fine details. I just want a big lot of food on the table now, fill them up and keep them going sort of thing. And then we’ve got the opposite end of that scale… Yeah, the big label readers and the big plant eaters, I suppose would be the most concerned”*

[P4, Ingredient Coordinator, Meal Kit Delivery].

## 4. Discussion

The current research has highlighted that there are low to high health literacy rates existing in domains where many of the public would consider such individuals to have high literacy. Low literacy B2C stakeholders had some knowledge of phytosanitation; however, this tended to be superficial and there was little knowledge about X-ray treatment and its potential advantages and greater safety. The terminology for X-ray and radiation is often confused, which supported the falsity that phytosanitary treatments were appraised as invasive. It is important that retailers in prospective markets be offered factual information from government regulators and independent science experts to highlight the benefits and science behind X-ray treatment, the details of the radiation source, and the greater safety of the method [32]. From a managerial perspective, access to and knowledge of X-ray treatment by central authorities such as government can support low literacy B2C stakeholders to have greater confidence in the technology. In contrast, poor knowledge may result in a purist narrative, where B2C stakeholders would keep consumers ‘in the dark’ as to phytosanitary information. Retailers are concerned that some consumers will see X-ray treatment/irradiation as potentially dangerous to long-term health. Resistance would be mainly limited to the health-conscious, who would avoid purchase [33].

For stakeholders with medium literacy, one key factor was the complexity of the information that influenced familiarity or naturalness. There were indications that more traditional retailers did not wish to highlight the sphere of biosecurity practices, preferring the practices to ‘fly under the radar’ to avoid damage to the familiarity of foods, caused by misunderstandings. Retailers believe most consumers are unaware of existing phytosanitary processes but would perceive any treatment as detrimental to the naturalness or familiarity of the produce. Larger traditional supermarket operations want to ensure customer safety, but in some cases the method used was based on the most effective and least disruptive method to ensure supply and consumption of fresh fruits and vegetables to price-sensitive customers. This results in some important implications, such as in the current research, the maintaining of the romance narrative for consumers. As narratives may be complex, there is a ‘bliss’ point in information that can impact how consumers may view technologies [34]. Raising awareness of the practices as part of the education could backfire and cause damage to the sector.

Finally, B2C stakeholders with high literacy, who followed sustainability-focused business models, had developed a good understanding of their consumer base and a sense of their own role in ensuring transparency. They saw education from a range of independent sources for consumers as important, and were confident that consumers would become self-informed [35]. Previous studies have concluded that consumers will view a reduction in the perceived risk once education on the benefits and reassurance about the safety of the technology is provided [36,37,38]. In practice, creating a consistent narrative about sustainability and the low-beam nature of the radiation, to alleviate concerns for those who seek more information, needs to be carefully designed by stakeholders.

## 5. Conclusions

The current study involved in-depth interviews with B2C (*n* = 12) retailers from sectors of the fresh produce industry who influence information about how foods, from a phytosanitary safety context, are promoted to consumers. Informing key retailers of the benefits and safety of X-ray treatment over other irradiation methods is a starting point. Overall, creating a consistent narrative about sustainability and the nature of the phytosanitary treatments needs to be considered, to alleviate concerns for those who seek more information. Ensuring that the ease of labelling practices for retailers will aid transparency for their consumers.

For consumers, it is a struggle to balance literacy of marketing health information behind the food products sold to them [17]. For key public stakeholders, such as retailers, there is often complexity around what to communicate to consumers [30]. Health literacy is key to support both individual micro-understanding of consumers, and also macro-level literacy in the food supply system.

The process of gaining acceptance for any new food technology is complex and multifaceted. The lag between expert knowledge of the benefits of X-ray treatment and wider public acceptance and confidence is likely to take time, but to gain greater social acceptability across the stakeholders, justifying the necessity for X-ray technology and its safety should be at the forefront of literacy. New Zealand consumers are increasingly focused on environmental and social sustainability concerns and there are clear benefits that will resonate with these personal values. The challenge is therefore to position X-ray food treatment in the minds of consumers as a modern and innovative technology promoting sustainability and advancement in food safety, whilst appeasing possible negative emotional reactions around unnaturalness and negative health associations with the general term of radiation.

Although participants were purposely recruited [23] from a narrow set of expertise, we acknowledge one limitation of the study is the sample size of 12 participants. We are confident in the number of participants as representative of their own views, because qualitative data analysis does not seek to generalize findings. However, future studies can expand the research scope to include a higher number of participants to generalize findings.

## Figures and Tables

**Table 1:** Participant Profiles.

Participant Number	Position	Categorization	Business Model/Philosophy	Buying Style
P1	Fresh produce buyer	Large supermarket chain	Traditional	Obtains for the store from a variety of suppliers
P2	Produce category manager	Premium supermarket chain	Traditional—premium	Obtains all produce from the markets and from growers directly
P3	Procurement manager	Meal kit delivery	New business model—sustainability approach	Uses large commercial producer wholesalers, buys from wholesalers and some growers directly
P4	Ingredient coordinator	Meal kit delivery	Customer-focused food delivery	Purchases through wholesale markets or direct from growers/packhouses
	Development kitchen and purchasing team			
P5	Small business owner	Food outlet	Small business, customer- and sustainability-focused	Food service companies. Specifies when needing organic goods, etc.
P6	Business owner	Organic stores	Sustainability-focused	Deals with growers and wholesalers
P7	General manager	Premium organic online supplier	New business model—social entrepreneurship	Buys direct from Auckland and Christchurch, and some organic wholesalers, small growers
P8	Retail manager	Food service	Social Entrepreneurship	Purchases through wholesalers at New Zealand markets
P9	Procurement and project manager	Food/juice outlet chain	New business model—sustainability approach	Sources through wholesalers via growers
P10	Small business owner	Catering	Traditional—basic	Sources via food service companies and supermarkets
P11	Business manager, fresh produce –head office	Large supermarket chain	Traditional	Deals with the brokers and growers directly to supply large supermarket stores
P12	General manager	Large business food service	Traditional	Purchases through wholesale markets or direct from growers/packhouses

**Table 2:** The Health Literacy Process in Phytosanitary Food Based Decision-Making (adapted from [17,31].

	Low Literacy	Medium Literacy	High Literacy
**Access and Comprehension** Ability to find and comprehend appropriate information	Superficial Associations	Trust in Regulations	Justified Safety
**Appraisals** Ability to interpret, filter, judge, and evaluate information	Invasiveness	Familiarity; Naturalness	Sustainability
**Application Narratives** Ability to apply and communicate information to position or influence a choice	The Purist Approach: Keep in the Dark	Maintaining the Romance	Full Transparency
